# Inhibition of *Plasmodium* sporogonic stages by ivermectin and other avermectins

**DOI:** 10.1186/s13071-019-3805-0

**Published:** 2019-11-21

**Authors:** Raquel Azevedo, António M. Mendes, Miguel Prudêncio

**Affiliations:** Instituto de Medicina Molecular, Faculdade de Medicina, Universidade de Lisboa, Avenida Professor Egas Moniz, 1649-028 Lisbon, Portugal

**Keywords:** *Plasmodium*, Malaria transmission, Avermectins, Ivermectin

## Abstract

**Background:**

The transmissible forms of *Plasmodium* parasites result from a process of sporogony that takes place inside their obligatory mosquito vector and culminates in the formation of mammalian-infective parasite forms. Ivermectin is a member of the avermectin family of endectocides, which has been proposed to inhibit malaria transmission due its insecticidal effect. However, it remains unclear whether ivermectin also exerts a direct action on the parasite’s blood and transmission stages.

**Methods:**

We employed a rodent model of infection to assess the impact of ivermectin treatment on *P. berghei* asexual and sexual blood forms *in vivo.* We then made use of a newly established luminescence-based methodology to evaluate the activity of ivermectin and other avermectins against the sporogonic stages of *P. berghei* parasites *in vitro* independent of their role on mosquito physiology.

**Results:**

Our results show that whereas ivermectin does not affect the parasite’s parasitemia, gametocytemia or exflagellation in the mammalian host, several members of the avermectin family of compounds exert a strong inhibitory effect on the generation and development of *P. berghei* oocysts.

**Conclusions:**

Our results shed light on the action of avermectins against *Plasmodium* transmission stages and highlight the potential of these compounds to help prevent the spread of malaria.

## Background

Malaria is a parasitic disease caused by *Plasmodium* parasites that are transmitted to their mammalian hosts by the bites of infected female *Anopheles* mosquitoes. In 2017, an estimated 435,000 deaths were attributed to malaria, 80% of which occurred in the regions of Africa and India and 61% of which in children under the age of 5. Although the number of malaria cases decreased from 2010, progress appears to have stalled, and a small increase in the global number of cases was actually observed in recent years [1].

*Plasmodium* sporozoites are injected by infected *Anopheles* mosquitoes into a mammalian host, homing to the liver, where they replicate into thousands of blood-infective merozoites [2].While most merozoites cyclically invade red blood cells, leading to disease [3], some differentiate into female and male gametocytes, which can be uptaken by mosquitoes [4]. Inside the mosquito midgut, gametocytes differentiate into female and male gametes that fuse forming zygotes [5]. In rodent *P. berghei* parasites, zygotes transform into ookinetes within 18–24 h, penetrating the mosquito’s midgut wall, and developing into oocysts 48 h after the blood meal [6, 7]. Over 10–14 days, oocysts mature in the basal lamina of the midgut wall, forming sporozoites which are eventually released and migrate to the mosquito salivary glands, where they remain ready to initiate a new mammalian infection [4, 8, 9].

The complexity of the *Plasmodium* parasite’s life cycle constitutes one of the biggest hurdles in the fight against malaria. So far, the most successful strategies to reduce the number of cases of malaria result from a combination of vector control strategies, and artemisinin combination therapies (ACT) that target the symptomatic blood stage of infection [1]. However, the emergence of mosquito resistance to insecticides and of parasite resistance to antimalarial drugs severely threaten the efficacy of such measures [1, 10]. Therefore, the identification of new compounds that have a broad spectrum of action, long half-life and strong inhibitory activity remains a priority in the fight against malaria.

Avermectins are a class of macrocyclic lactones with insecticidal and antiparasitic properties. They are the most effective and well-developed class of endectocides, and are active against both endo- and ectoparasites [11]. Ivermectin, the best studied semi-synthetic derivate of avermectin, has been considered one of the most successful discoveries in the fight against infections caused by roundworm parasites [12]. Mass drug administration (MDA) of ivermectin in Africa and Latin America led to a reduction of onchocerciasis, as well as of lymphatic filariasis and scabies, which are also endemic in India and Southeast Asia [12–14]. The impact of ivermectin on insect vectors [12, 13], in particular its activity against *Anopheles* mosquitoes [15, 16] prompted the investigation of its action against *Plasmodium* parasites, towards harnessing its potential use as an integrated tool for malaria control [17, 18]. We have recently reported on ivermectin activity against *Plasmodium* liver stages *in vivo* [19]. However, discrepant results have emerged from several other studies aimed at assessing the impact of ivermectin on the blood and mosquito stages of the *Plasmodium* life-cycle [20–25]. Whereas results obtained by Nasveld et al. [20] indicated that ivermectin displays very low activity against *P. falciparum* blood stages *in vitro*, Panchal et al. [22] suggested that ivermectin inhibits the parasite’s blood stage development by blocking nucleo-cytoplasmic shuttling of *P. falciparum* signal recognition particle (SRP) components. This is in agreement with a recent study that demonstrated an impairment of sexual and asexual stages of *P. falciparum* development *in vitro* [25]. On the other hand, ivermectin was reported to reduce oocyst prevalence and intensity in different mosquito species infected with *P. falciparum* [21], contrary to reports by Kobylinski et al. [23] and Pinilla et al. [24], who did not observe a reduction on oocyst intensity, i.e. the number of oocysts per mosquito, but rather a decrease on oocyst prevalence (the proportion of mosquitoes harbouring oocysts), for *P. vivax* and *P. falciparum*.

The present study aimed to clarify the impact of ivermectin on *P. berghei* blood stages *in vivo*, as well as to assess the impact of ivermectin and other avermectins on the parasite’s mosquito stages *in vitro*.

## Methods

### Experimental animals and *P. berghei* ANKA reference lines

Male BALB/cByJ mice (6–8 weeks-old) purchased from Charles River Laboratories Inc. (Lyon, France) were used. Two parasite lines were employed in the experimental work, a transgenic parasite line termed *Pb*CSGFP-Luc (RMgm-152),which expresses the fusion gene *gfp-luc* under the control of the *csp* promotor (PBANKA_0403200) integrated into the silent *230p* gene locus (PBANKA_0306000) [26], and the transgenic parasite line *Pb*Fluo-frmg (RMgm-164), which expresses GFP under control of the ‘male gametocyte-specific’ promoter of PB000791.03.0 (dynein heavy chain, putative) and RFP under the control of the ‘female gametocyte-specific’ promoter PB000504.02.0 (LCCL domain-containing protein CCP2). The *rfp* and *gfp* genes are integrated into the genome in the *230p* locus (PBANKA_0306000) [27].

### Ookinete production

*Plasmodium berghei* ANKA expressing green fluorescent protein (GFP) and luciferase under the control of the circumsporozoite protein (CSP) promoter (line 784cl1, RMgm-152, *Pb*CSPGFP-Luc) was maintained in *Anopheles stephensi* mosquitoes and BALB/cByJ mice. To maintain gametocyte infectivity, only up to six passages of parasites from infected to naïve mice were performed. Ookinete *in vitro* production was performed as previously described [26]. Briefly, BALB/cByJ mice were treated with 0.1 ml phenylhydrazine (25 mg/ml) 3 days prior to infection with 10^7^ *P. berghei-*infected red blood cells (iRBC) obtained from a donor mouse. On the third day after infection, gametocytemia was monitored by light microscopy for the presence of exflagellating gametocytes in ookinete medium (1:4 dilution). Blood collected by heart puncture was pooled from 2 mice and washed with RPMI at 37 °C, followed by centrifugation at 1100×*g* for 10 min at 37 °C. After washing, 5 μl of blood containing exflagellating gametocytes was mixed with medium supplemented for ookinete formation [RPMI1640 (Sigma-Aldrich, Saint Louis, USA), 25 mM HEPES, 0.4 mM hypoxanthine, 100 mM xanthurenic acid (85570, Fluka, Saint Gallen, Switzerland), 10% FBS (pH 7.6)] in a final volume of 200 μl, and cultured in 96-well plates for 24 h at 19 °C. Additionally, blood containing exflagellating gametocytes was mixed with the ookinete medium in 1:20 ratio and cultured in T75 flasks for 22–24 h at 19 °C. Following incubation, ookinete enrichment was performed as previously described [26], with some modifications. Briefly, cultured blood was collected, and erythrocytes were lysed for 15 min on ice with 30 volumes of ice-cold 0.17 M ammonium chloride. Lysed erythrocytes were removed by washing with RPMI, and ookinetes were purified by centrifugation on a 63% Nycodenz cushion at 650×*g* at 4 °C for 30 min. Following centrifugation, the ookinete-containing interface was collected, washed in ice-cold RPMI and resuspended in 0.5–1.0 ml of oocyst medium.

### Oocyst cultures

Purified ookinetes were seeded with *Drosophila melanogaster* S2 cells (*Drosophila* Genomics Resource Center, Bloomington, USA) in a 1:10 ratio (10^4^ ookinetes and 10^5^ S2 cells) in Schneider’s medium (S0146, Sigma-Aldrich) supplemented with 15% FBS, penicillin/streptomycin (50 U/ml, 50 µg/ml) and gentamicin (50 µg/ml) to promote oocyst development. Oocysts were co-cultured with *D. melanogaster* S2 cells in flat bottom 96-well plates (Corning, New York, USA) for up to 15 days at 19 °C. One-quarter of the medium was changed 3 times per week (every 48 to 72 h), and 10^5^ S2 cells were added once per week. In parallel, S2 cells were maintained at 27 °C in Schneider’s medium (S0146, Sigma-Aldrich) supplemented with 10% FBS and penicillin/streptomycin (50 U/ml, 50 µg/ml).

### Bioluminescence assay

A bioluminescence assay was used to assess the development of the mosquito stages of *Pb*CSGFP-Luc. In order to evaluate the effect of compounds on the development of the parasite’s mosquito stages, samples were collected at 3 different time points to determine the effect on ookinete and oocyst formation, and oocyst maturation, as previously described [26]. The bioluminescence assay was performed using the Firefly Luciferase Assay Kit (Biotium, Hayward, USA) according to the manufacturer’s instructions, with some modifications. Briefly, the whole well contents were collected and spun down, washed with PBS, frozen in 50 µl of lysis buffer (1:5 ratio) and stored at − 20 °C until further use. Collected samples were lysed and 30 µl of the resulting supernatant were transferred into white 96-well plates. Fifty µl of D-luciferin in Firefly luciferase assay buffer (1:50 ratio) were added to the samples and parasite load was determined by measuring luminescence intensity using a microplate reader (Tecan Infinite M200, Zurich, Switzerland).

### Evaluation of the *in vivo* activity of ivermectin

In order to assess the *in vivo* activity of Iv, five BALB/cByJ (Charles River) mice per experimental group were infected by intraperitoneal injection of 10^7^ *P. berghei* Fluo-frmg-infected red blood cells. Parasitaemia and gametocyteamia were measured the following days by flow cytometry analysis of 4 µl of tail blood. Blood was collected in 200 µl of PBS and 100 µl stored at 4 °C, while the remaining was transferred to 100 µl of PBS containing 1.25 mM of red fluorescent nucleic acid stain Syto®61 (Thermo Fisher Scientific, Waltham, USA) and incubated for 20 min at room temperature in the dark. The samples were analysed on a LSRFortessa X-20 flow cytometer (Becton, Dickinson and Co., New Jersey, USA). Female and male gametocytes were gated based on the analysis of GFP and Red Fluorescence Protein (RFP) fluorescence, and parasitemia was estimated based on the analysis of Syto®61 and forward scatter. The positive cell population was determined by comparison of infected blood samples with an uninfected blood sample. Results were then analysed with the FlowJo^TM^ Software (Version 10, FlowJo^TM^ Software, Ashland, USA). Exflagellation was also monitored every day, until parasitemia reached 3% by microscopy analysis. To this end, 2.5 µl of tail blood were collected and mounted on a glass slide, and 8 min later the number of exflagellation events present in 4 independent fields of vision at 40× were determined. When parasitemia reached up to 3%, either DMSO or ivermectin were administered by oral gavage, at a concentration of 5 mg/kg, to five mice of each experimental group. Parasitemia, gametocytemia and exflagellation were monitored for 3 days after treatment, following which the mice were euthanized, and the experiment was terminated.

### Evaluation of the *in vitro* activity of avermectin compounds

The effect of eprinomectin, abamectin, ivermectin, moxidectin, doramectin and emamectin on the *Plasmodium* mosquito stages was evaluated as previously described [26]. The compounds were dissolved in dimethyl sulfoxide (DMSO) and the amount of DMSO equivalent to that present in the highest compound concentration was used as a control. The effect of 10 µM of each compound was assessed on ookinetes, and on oocyst development and maturation. Briefly, after 1 h of incubation, the compounds were added to 5 µl of infected blood cultures, and the bioluminescence intensity of the parasites on the ookinete cultures was assessed 24 h later. To assess the effect of the compounds on oocyst development and maturation, compounds were mixed with the mature ookinetes, and parasites collected after 3 days of culture or compounds were added to the oocyst culture after 3 days of culture, and parasites collected 15 days later.

Compound concentration resulting in 50% inhibition (IC_50_) for oocyst growth and maturation were estimated for eprinomectin, abamectin, ivermectin, moxidectin, doramectin and emamectin (assayed at 0.05, 0.5, 1, 5, 10, 25 and 50 µM) by nonlinear regression analysis.

### Evaluation of avermectin compounds’ *in vitro* cytotoxicity

Compounds were screened for their *in vitro* cytotoxicity against *D. melanogaster* S2 cells, using the AlamarBlue® assay (Invitrogen, Carlsbad, USA). This assay allows to measure metabolic activity based on a fluoremetric/colorimetric indicator [28]. To assess the effect of the compounds on cell development, *Drosophila melanogaster* S2 cells were seeded in a 1:10 ratio (10^5^ S2 cells) in Schneider’s medium (S0146, Sigma-Aldrich) supplemented with 15% FBS, penicillin/streptomycin (50 U/ml, 50 µg/ml) and gentamicin (50 µg/ml). All the above-mentioned compounds were added to the S2 cell cultures to a final concentration of 10 µM and the amount of DMSO equivalent to that present in the highest compound concentration was used as a control. Cultures were maintained for 7 days and one-quarter of the medium was changed 3 times per week every 48 to 72 h. Samples were collected every day by removing 120 µl of medium and adding 80 µl of AlamarBlue previously diluted in Schneider’s medium (1:10 dilution) to each well. The suspension was transferred to a 96 well flat bottom plate and incubated for one and a half hours at 37 °C. Fluorescence intensity was then measured using a microplate reader (Tecan Infinite M200) at 530 nm excitation wavelength/590 nm emission wavelength to determine cell viability.

### Statistical analysis

Data on the assessment of the compounds’ effect *in vitro* were analysed using the Kruskal-Wallis test. Data on the compounds’ effect on parasitemia, gametocytemia and exflagellation *in vivo* were analysed employing non-linear regression analysis. Results were considered significant for *P*-values < 0.05. Nonlinear regression analysis was employed to fit the normalized results of the dose-response curves for IC_50_ determination. All statistical tests were performed by GraphPad Prism (version 6.00, GraphPad Software, La Jolla, USA).

## Results

### *In vivo* activity of ivermectin against *P. berghei* blood stages

**Fig. 1:**
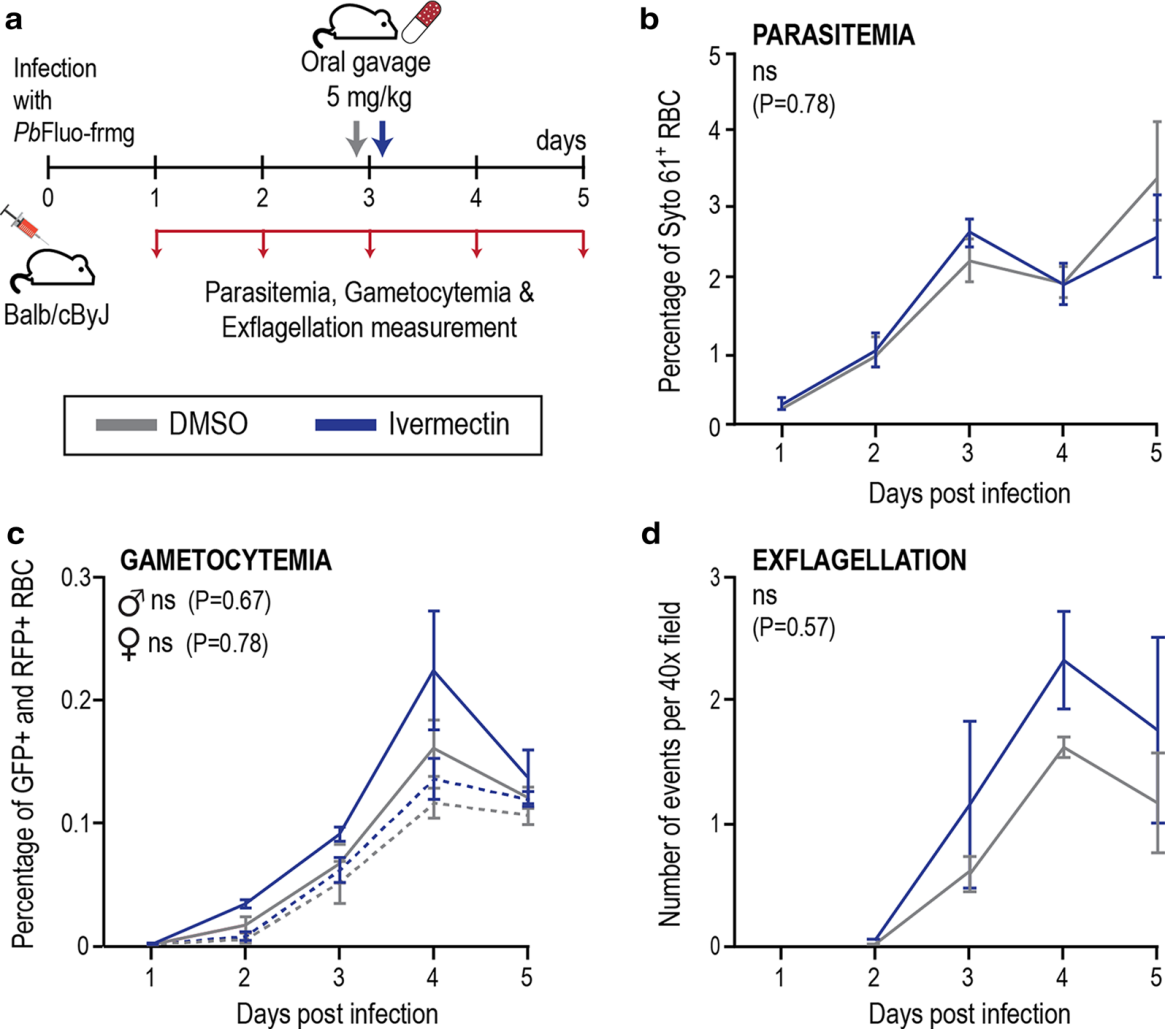
Ivermectin activity against *P. berghei* asexual and sexual blood stages in the mammalian host. **a** Schematics of assessment of *in vivo* compound activity on parasitemia, gametocytemia and exflagellation of mice treated with ivermectin. **b** *In vivo* activity of ivermectin on parasitemia of mice treated by a single oral dosage of ivermectin. Results are a representation of Syto 61 positive events on flow cytometry analysis and are expressed as the mean of parasitemia values (percentage of infected red blood cells) ± standard deviation (SD). **c** *In vivo* activity of ivermectin on female and male gametocytemia of mice treated by a single oral dosage of ivermectin. Female gametocytemia is represented by a dashed line and male gametocytemia by a solid line. Female and male gametocytes were identified by flow cytometry analysis of RFP^+^ or GFP^+^ events, respectively. Results are expressed as the mean of gametocytemia values (percentage of gametocytes) ± SD. **d** *In vivo* activity of ivermectin on the number of exflagellation events per 40× microscopic field of mice treated by a single oral dosage of selected drug. Results are expressed as the mean of observed exflagellation events ± SD. *Abbreviations*: ns, non-significant

A mouse model of infection was employed to evaluate the effect of ivermectin on *P. berghei* sexual and asexual blood stage forms *in vivo*, as described (Fig. 1a). Our results show no statistically significant differences between untreated controls and ivermectin-treated mice regarding overall parasitemia (*F*_(1, 26)_ = 0.074, *P* = 0.78), percentage of the parasite’s male and female gametocytemia (*F*_(1, 26)_ = 0.18, *P* = 0.67 and *F*_(1, 26)_ = 0.079, *P* = 0.78, respectively), and exflagellation events (*F*_(1, 26)_ = 0.33, *P* = 0.57) up to 5 days after infection (Fig. 1b–d). These data suggest that, at the tested dosage, ivermectin does not appear to significantly inhibit *Plasmodium* asexual and sexual forms in the blood.

### *In vitro* activity of avermectins against *P. berghei* sporogonic development

**Fig. 2:**
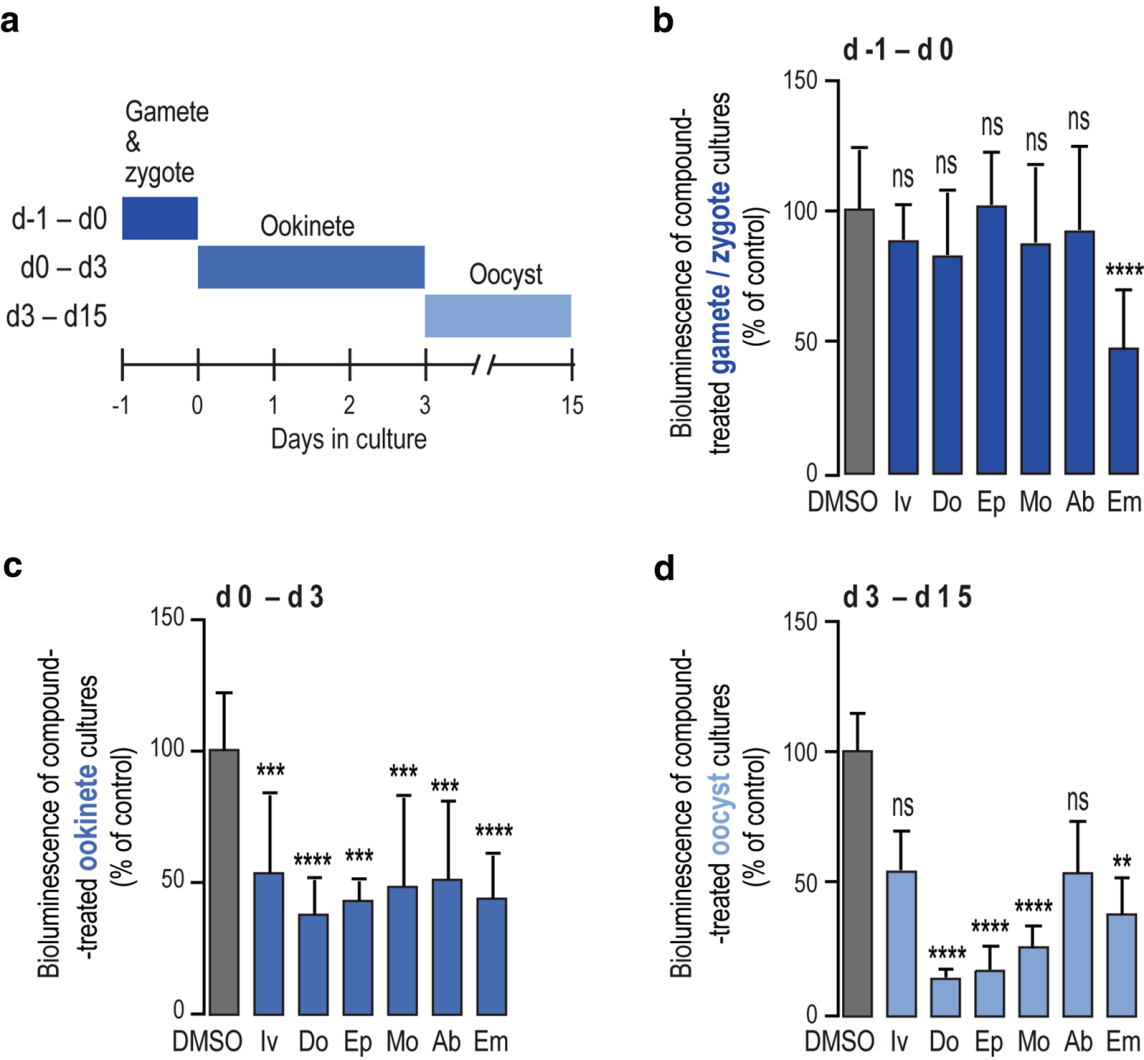
Activity of avermectins against *Plasmodium* mosquito stages *in vitro*. **a** Schematics of the progress of the parasite culturing process, highlighting the different schedules of compound treatment employed. **b** Assessment of compound effects on ookinete formation, expressed as the percentage of inhibition of *P. berghei* ookinete formation. **c** *In vitro* activity of selected compounds against oocyst formation. **d** *In vitro* activity of selected compounds on oocyst development. A total of 6 compounds were screened at a concentration of 10 μM: Iv; Do, Ep, Mo, Ab and Em. Bars correspond to RLU measurements represented as the percentage of RLU of the DMSO control. Results are expressed as the mean + SD. ****P < 0.0001, ***P < 0.001, **P < 0.01. *Abbreviations*: Iv, ivermectin; Do, doramectin; Ep, eprinomectin; Mo, moxidectin; Ab, abamectin; Em, emamectin

We then sought to clarify whether the proposed transmission-reducing activity of ivermectin would include a direct action of the drug on the parasite’s transmission stages in the mosquito or if it would result solely from its effect on the mosquito. Given the marked structural similarities between avermectins, the effect an additional 5 compounds of this family, doramectin, moxidectin, abamectin, emamectin, and eprinomectin on the parasite’s sporogonic stages was also evaluated, as described (Fig. 2a).

Our assessment of the compounds’ effect on the transformation of gametes/zygote into ookinetes, showed that whereas emamectin inhibited parasite differentiation by approximately 47%, ivermectin and the remaining avermectins had a very small and non-significant impact on the formation of ookinetes (Fig. 2b). The effect of avermectins on oocyst formation and growth was subsequently assessed. At 10 µM, both these processes are inhibited by at least 50% by all compounds (Fig. 2c, d). Half-maximal inhibitory concentrations (IC_50_) for oocyst formation by the compounds under evaluation were then determined and found to range from ~ 5.7 µM to ~ 11.6 µM, with eprinomectin, doramectin and emamectin displaying the greatest potency against this early stage of parasite sporogony (Table 1, Additional file 1: Figure S1a). Their IC_50_ values for oocyst growth varied between ~ 4.3 µM and ~ 9.3 µM (Table 1, Additional file 1: Figure S1b), with eprinomectin, doramectin and moxidectin standing out as the three compounds that most potently inhibited oocyst development (Table 1). Since oocysts are co-cultured in the presence of *Drosophila* S2 cells, we evaluated the latter’s viability for up to 7 days in the presence of each of the compounds under study (Additional file 1: Figure S2). Of note, these results indicate that emamectin displays some cytotoxicity against S2 cells, which might suggest a moderate overestimation of this compound’s activity against developing oocysts.

**Table 1:** IC_50_ of avermectins against oocyst formation and maturation *in vitro*

Compound	Day0–Day3 (µM)		Day3–Day15 (µM)	
	IC_50_	SD	IC_50_	SD
Eprinomectin	5.70	2.31	4.59	2.26
Doramectin	7.08	1.91	4.32	1.76
Emamectin	7.31	2.45	7.36	1.50
Abamectin	8.77	4.38	7.93	4.14
Moxidectin	10.85	8.56	5.49	1.00
Ivermectin	11.58	0.44	9.32	1.64

*Notes*: Compound concentration resulting in 50% inhibition (IC50) for oocyst growth and maturation were calculated for Ep, Do, Em, Ab, Mo, and Iv (assayed at 0.05, 0.5, 1, 5, 10, 25 and 50 µM). Results are expressed as the mean ± SD

*Abbreviations:* Ep, eprinomectin; Do, doramectin; Em, emamectin; Ab, abamectin; Mo, moxidectin; Iv, ivermectin

## Discussion

In the present study, we aimed to assess the impact of ivermectin on the blood stages of *P. berghei in vivo*, as well as the effect of several avermectins on the parasite’s mosquito stages *in vitro*. Our results show that ivermectin is not active against *Plasmodium* asexual and sexual blood forms in a mouse model. However, both ivermectin and other members of the avermectin family strongly inhibited parasite sporogony, at IC_50_ values consistent with those reported for the antibiotic thiostrepton and the antimalarial pyronaridine, whose impact on sporogony has been demonstrated [29].

In light of their versatility as antiparasitic and insecticidal compounds, avermectins and, particularly ivermectin, have been considered as potential aids in the fight against malaria [16, 18, 19, 21–23]. Of note, the use of ivermectin in MDA to treat other neglected tropical diseases in malaria-endemic regions has led to the investigation of its potential to block malaria transmission [18, 30, 31]. A recent study showed that a 3 weekly MDA of ivermectin in African villages reduced the incidence of uncomplicated malaria episodes among children, which the authors attributed to the drug’s mosquitocidal effect [18]. However, it should be noted that the results of this study have recently been questioned on the basis of the statistical methods employed [32].

Nevertheless, the issue of whether the impact of ivermectin stems solely from its impact on mosquitoes or, additionally, results from a combination of its insecticidal activity and of its ability to inhibit the parasite’s blood and mosquito stages remains unresolved. In fact, while the inhibitory effect of ivermectin against the liver stages of *Plasmodium* parasites has been demonstrated [19], the evaluation of its impact against the blood [20, 22, 25] and sporogonic [15, 23, 24] stages of the parasite’s life-cycle has yielded contradictory results [15, 20, 22–25].

In invertebrates, ivermectin interacts with the glutamate-gated chloride channels in neuronal and neuromuscular tissues [33–35] and may also act on the γ-aminobutyric acid-gated chloride channels [36–38]. However, neither of these molecular targets is present in *P. falciparum*, which might explain the lack of ivermectin *in vitro* effect against *P. falciparum* blood stages [20]. Contradictorily, ivermectin was reported to lead to the arrest the development of *P. falciparum* blood stages by inhibiting the nuclear import of SRP polypeptides, thus arresting parasite growth [22]. The sporontocidal activity of ivermectin against *P. vivax* in *An. darlingi* led to a reduction of oocyst prevalence, but not of their intensity [24], similarly to what has been observed for *P. falciparum* in *An. gambiae* [16]. This contradicts previous studies suggesting that ivermectin reduces oocyst prevalence and intensity in *An. dirus* and *An. minimus* [21]. Although the mechanism of action of ivermectin against the sporogonic stages of *Plasmodium* parasites remains to be elucidated, these observations suggest that the compound may act on the mosquito midgut physiology, preventing parasite establishment [23]. As such, the different results obtained experimentally in previous studies might result from differences in insect biology.

Our study sheds a new light on these controversial issues and helps clarify whether ivermectin exerts an impact on the blood and/or on the mosquito stages of *Plasmodium* parasites. Our *in vivo* investigation revealed that treatment of infected mice with 5 mg/kg of ivermectin had no impact on parasitemia, indicating an absence of activity of this compound against the parasite’s asexual forms in the blood, in accordance with Nasveld et al. [20] for *P. falciparum*.

To further clarify whether a direct drug effect on *Plasmodium* mosquito stages contributes to transmission blocking, ivermectin and other avermectins were employed in a mosquito-free *in vitro* assay. Our results indicate that while these compounds exert little or no activity against ookinete formation, they efficiently inhibit the parasite’s sporogonic stages after fertilization, most effectively targeting late stage oocysts. Further investigation on the mechanism of action of avermectins is required in order to fully clarify the exact targets of their activity against *Plasmodium* sporogony.

## Conclusions

Our results suggest that the impact of ivermectin on *Plasmodium* transmission does not result from an inhibition of *Plasmodium* spp. transmissible forms in the mammalian host, and stems solely from its effect during the mosquito stage of infection. Our data show that the transition from gamete/zygote to ookinete is highly resistant to avermectins, and that the oocyst is the most vulnerable stage of the parasite’s sporogonic cycle to treatment with avermectins. Collectively, these observations support the notion that, besides their mosquitocidal effect, avermectins may also directly target the parasite’s sporogonic stages, which likely contributes to transmission-blocking activity. Our results lend further support to the use of avermectins for MDA as a tool for malaria control in endemic regions, and suggest that the inclusion of members of this family of avermectin compounds besides ivermectin in these interventions should be considered.

## Supplementary information

**Additional file 1: Figure S1.** Dose-response of avermectins against *Plasmodium* sporogonic stages. **a** Representative curves of avermectins effect resulting in 50% inhibition (IC_50_) of oocyst formation. Curves are presented for Ep, Do, Em, Ab, Mo and Iv (assayed at 0.05, 0.5, 1, 5, 10, 25 and 50 µM). Results are expressed as the mean ± standard deviation (SD). **b** Representative curves of avermectinʼs effect resulting in 50% inhibition (IC_50_) of oocyst maturation. Curves are presented for Ep, Do, Em, Ab, Mo and Iv (assayed at 0.05, 0.5, 1, 5, 10, 25 and 50 µM). Results are expressed as the mean ± SD. **Figure S2**. Evaluation of avermectinʼs cytotoxicity on S2 cells. Determination of cell viability in a time course of 7 days by the AlamarBlue® assay. Results are normalized to the DMSO control and expressed as the mean ± SD. *Abbreviations:* DMSO, dimethyl sulfoxide; Ep, eprinomectin; Do, doramectin; Em, emamectin; Ab, abamectin; Mo, moxidectin; Iv, ivermectin.

## Data Availability

All data generated or analysed during this study are included in this published article and its additional file.

## Abbreviations

CSP: circumsporozoite protein
DMSO: dimethyl sulfoxide
FBS: foetal bovine serum
GFP: green fluorescent protein
IC_50_: concentration that inhibits parasite load by 50%
iRBC: infected red blood cells
Luc: luciferase
MDA: mass drug administration
RFP: red fluorescent protein
RPMI: Roswell Park Memorial Institute
