# Supplementary material for: Inhibition of Plasmodium sporogonic stages by ivermectin and other avermectins
Source: Parasit Vectors. 2019 Nov 21;12:549. doi: 10.1186/s13071-019-3805-0 (PMC6873674; doi:10.1186/s13071-019-3805-0)

**Additional file 1**

**Inhibition of *Plasmodium* sporogonic stages by ivermectin and other avermectins**

by Raquel Azevedo, António M. Mendes, Miguel Prudêncio

**Figure S1.** Dose-response of avermectins against *Plasmodium* sporogonic stages. **a** Representative curves of avermectins effect resulting in 50% inhibition (IC_50_) of oocyst formation. Curves are presented for Ep, Do, Em, Ab, Mo and Iv (assayed at 0.05, 0.5, 1, 5, 10, 25 and 50 µM). Results are expressed as the mean ± standard deviation, SD. **b** Representative curves of avermectinʼs effect resulting in 50% inhibition (IC_50_) of oocyst maturation. Curves are presented for Ep, Do, Em, Ab, Mo and Iv (assayed at 0.05, 0.5, 1, 5, 10, 25 and 50 µM). Results are expressed as the mean ± SD.

*Abbreviations:* Ep, eprinomectin; Do, doramectin; Em, emamectin; Ab, abamectin; Mo, moxidectin; Iv, ivermectin.


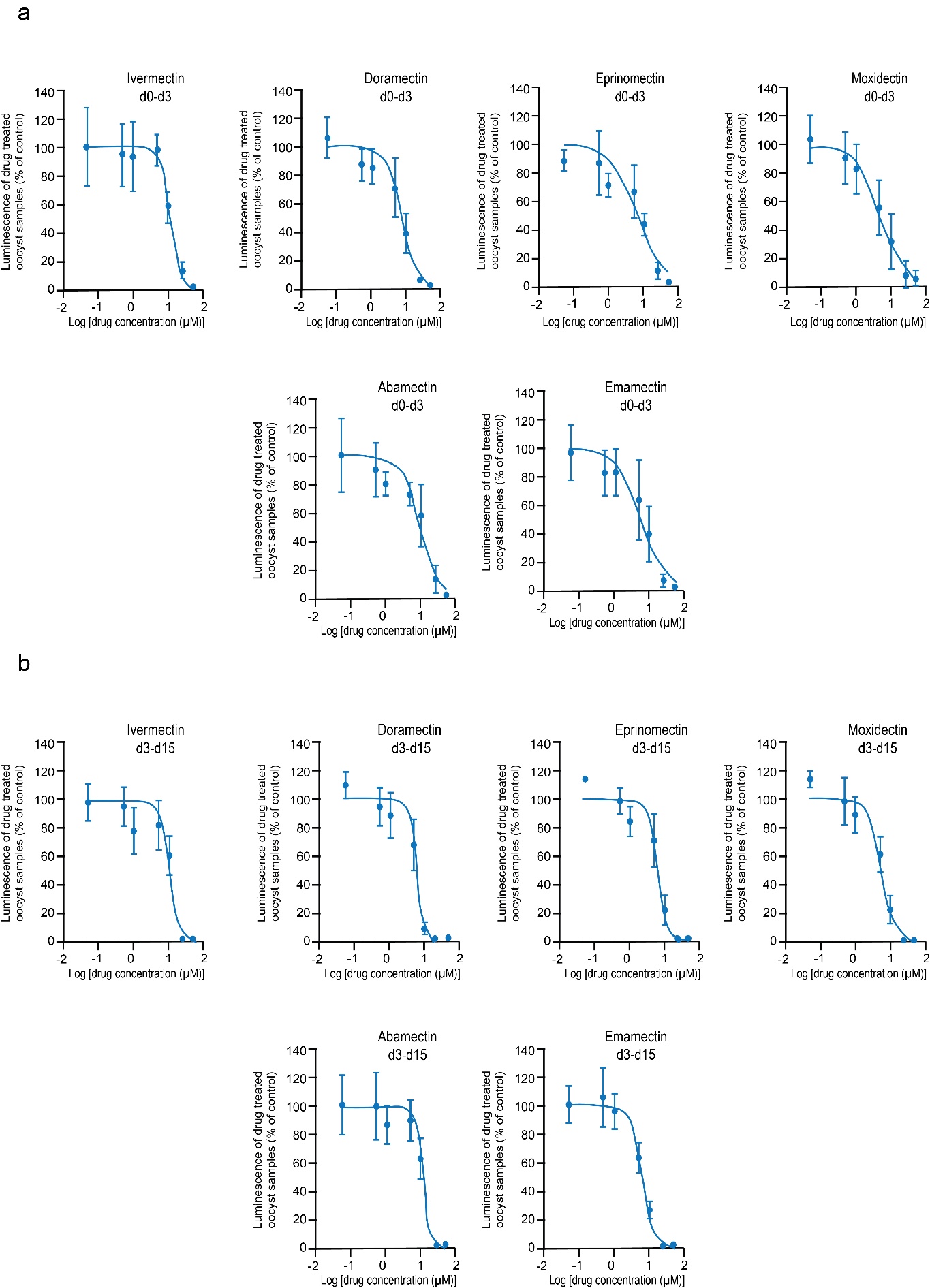


**Figure S2.** Evaluation of avermectinʼs cytotoxicity on S2 cells. Determination of cell viability in a time course of 7 days by the AlamarBlue® assay. Results are normalized to the DMSO control and expressed as the mean ± standard deviation, SD.

*Abbreviations:* DMSO, dimethyl sulfoxide; Ep, eprinomectin; Do, doramectin; Em, emamectin; Ab, abamectin; Mo, moxidectin; Iv, ivermectin.


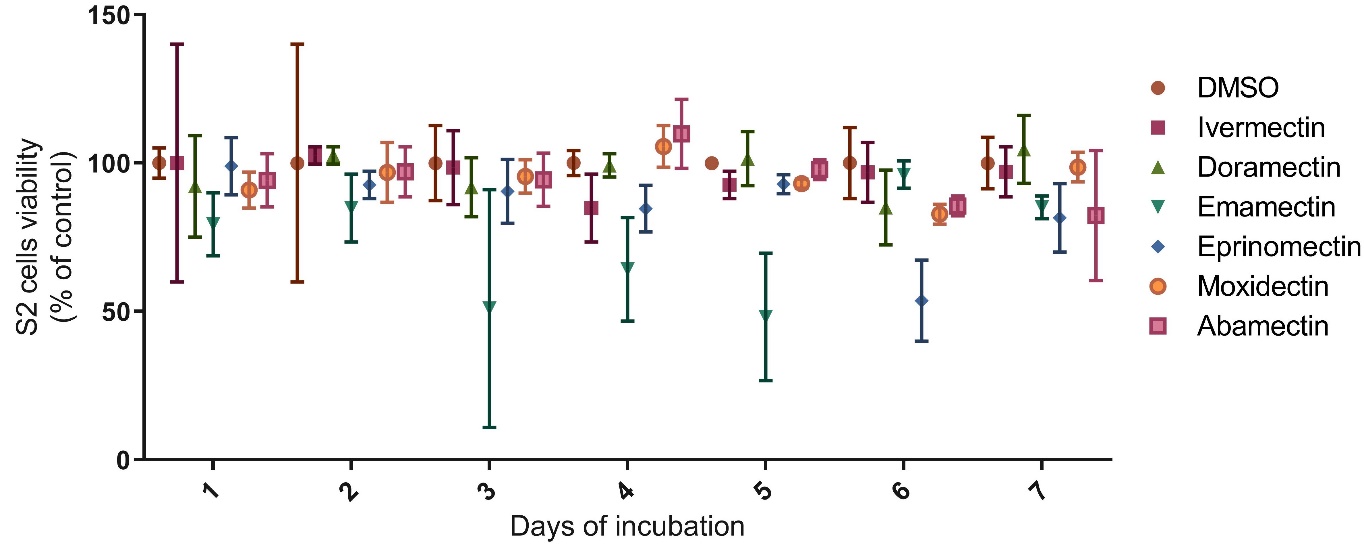

Supplement: Supplementary file 1 — Additional file 1: Figure S1. Dose-response of avermectins against Plasmodium sporogonic stages. a Representative curves of avermectins effect resulting in 50% inhibition (IC50) of oocyst formation. Curves are presented for Ep, Do, Em, Ab, Mo and Iv (assayed at 0.05, 0.5, 1, 5, 10, 25 and 50 µM). Results are expressed as the mean ± standard deviation (SD). b Representative curves of avermectinʼs effect resulting in 50% inhibition (IC50) of oocyst maturation. Curves are presented for Ep, Do, Em, Ab, Mo and Iv (assayed at 0.05, 0.5, 1, 5, 10, 25 and 50 µM). Results are expressed as the mean ± SD. Figure S2. Evaluation of avermectinʼs cytotoxicity on S2 cells. Determination of cell viability in a time course of 7 days by the AlamarBlue® assay. Results are normalized to the DMSO control and expressed as the mean ± SD. Abbreviations: DMSO, dimethyl sulfoxide; Ep, eprinomectin; Do, doramectin; Em, emamectin; Ab, abamectin; Mo, moxidectin; Iv, ivermectin. [file 13071_2019_3805_MOESM1_ESM.docx]
